# Terminology matters: defining the esports athlete

**DOI:** 10.3389/fspor.2023.1232028

**Published:** 2023-08-25

**Authors:** Kabir Bubna, Michael G. Trotter, Remco Polman, Dylan R. Poulus

**Affiliations:** ^1^ The International Federation of Esports Coaches (IFoEC), London, United Kingdom; ^2^ Department of Psychology, Umeå University, Umeå, Sweden; ^3^ Institute of Health and Wellbeing, Federation University Australia, Berwick, VIC, Australia; ^4^ Physical Activity, Sport and Exercise Research Theme, Faculty of Health, Southern Cross University, Gold Coast, QLD, Australia

**Keywords:** esports, esports athletes, terminology, definitions, gamer

## 1. Introduction

There has been increasing interest in esports from participation, spectator, and academic perspectives. Concerning the latter, there have been discussions about whether esports can be classified as a sport and its definition (1). A range of academic disciplines have started investigating the esports industry [e.g., Business, Sport Science, Law, Cognitive Science; see (2)], and have provided several differing definitions for esports. We believe that within these definitions, there is a need to consistently distinguish between individuals who play the various esports titles as a form of leisure compared to those who play them competitively. A commonly adopted definition of esports was proposed by Pedraza-Ramirez et al. (3), who view esports as “the casual or organized playing of competitive computer games facilitated through in-game ranking systems or organized competitions” (p. 6).

Studies examining whether esports can be called “sports” provide compelling evidence that this is the case. Campbell et al. (4) suggest that sports sit on a continuum between those which are predominantly physical (e.g., weightlifting) and those which are more cognitively focused (e.g., chess). Esports generally meet the criteria of various definitions of sports (5). The clear distinction between the two domains (sports and esports) lies in the fact that performance is enacted through different mediums, most esports do not adhere to the definition of “sport” presented by Connor (6) “a sport is a game involving physical exertion” (pg 15) whereas, in esports performance is conducted through electronic systems where the use controls an avatar. However, esports and sports share similarities in the sense, that in most cases there are organised teams, there is an element of competition (i.e., winners and losers), and with the professionalisation of esports, counters hours are dedicated to training and refining skill for competetitve success (7). Hence, many of the characteristics attributed to sports also apply to esports (2, 8).

Generally, the term “athletes” refers to those who participate in sports. However, when referring to competitors of specific sports (especially team sports), athletes are also referred to as players (e.g., football, hockey players). We will argue in this paper that a similar hierarchical structure of naming individuals engaging in esports should be adopted in esports research to facilitate clarity on the sample and enhance comparisons across (e)sports.

As esports are widely considered sports, we seek to establish clarity about which term should be used regarding individuals who participate within the vast esports ecosystem. Having consistent terminology that differentiates between the recreational gamer and competitive gamer is an important issue for several reasons. Firstly, this reduces the confusion that might occur across the esports literature. Secondly, it allows comparisons with sports and exercise literature. Thirdly, it allows for a more accurate description of the different groupings of esports participants.

Developing a clearer understanding of how we define those who play video games competitively will allow more targeted research and comparisons. For example, this would allow for research to explore how esports athletes differ from games in terms of health behaviours like physical exercise, nutritional practices, smoking, and drinking as well as psychosocial characteristics like social support and self-regulation.

## 2. Key terminology within esports literature

Across the range of literature, there is a clear incongruence in the terms used to describe esports athletes (see Table 1). Terms such as esports player, esports athlete, gamer, and video game players can all describe the individuals who interact within the vast esports ecosystem in any competitive manner (i.e., ranked play or structured competition). At times, when referring to the more formal and organized categories, terms may be prefaced, such as “elite” e-athletes, “professional” video gamers, and “competitive” esports players. Due to the nature of esports, and following the definition provided by Perdraza-Ramierez et al. (3), all esports feature some in-game or external ranking system that categorizes players into various rankings indicative of their skill. For example, League of Legends (LoL) features a ranking system that spans from Iron (the lowest) to Challenger (the highest). With a majority of the player base placed between Silver and Gold, and with ∼1% of the participants populating the Master, Grandmaster and Challenger tiers (24). However, not everyone within these tiers participates for a ranking or within a structured and competitive system. Many align themselves with content creation or streaming, providing high-quality live or on-demand entertainment for a larger audience. Cases like this are not exclusive to LoL but feature across almost all prominent esports titles, further highlighting the need to create clear terminology between athlete, entertainer, and player to avoid future ambiguity as the scientific literature within esports develops.

**Table 1:** A range of terminology used in existing literature pertaining to esports.

Discipline	Paper/Authors	Terminology used	Definition
Business	Reitman et al., (2)	Video game players; cybersport	N/A
	Huttermann et al., (9)	Esports athletes or players	N/A
Psychology	Trotter et al., (10)	Esports Athletes (e-athletes)	“We define e-athletes as those who play an esport and have an official ranking for that esport.”
	Poulus et al., (11)	“Elite” esports athletes	N/A
	Leis et al., (12)	Esports Players; “Professional” Esports Players	N/A
	Smith et al., (13)	Players; Gamers; Elite esports competitors	N/A
Physiology	DiFrancisco-Donoghue et al., (14)	Esports players	N/A
	McNulty et al., (15)	Esports Players; Professional League of Legends Players	N/A
	Kari & Karhulahti (16)	E-athletes; professional e-athletes	“As defined by their team contracts or achievements in international tournaments”.
Injury & Health	McGee & Ho., (17)	Esports competitors; Professional players	N/A
	McGee et al., (18)	Esports competitors; competitive gamers	N/A
	Trotter et al., (19)	Esports player; Esports athlete	Esports are video games specifically designed with competition in mind. Video games, on the other hand, are a leisure activity designed to entertain.
Computer Sciences	Tȕrkay et al., (20)	Collegiate Players; Esports Players	N/A
	Khormov et al., (21)	Esports Athletes; Esports Players	“In this research, we define an athlete as a professional player with a work contract with a professional eSports team… A player is a person without the eSports contract while having relevant game skills or status.”
Coaching	Watson et al., (22)	Esports players	N/A
	Bubna et al., (23)	Esports Athletes; professional players; professional gamers	N/A

## 3. Current research and definitions

As mentioned previously, many academic domains have started to pay attention to the world of esports. In doing so, they have created a range of definitions and terminology. Although not an exhaustive list, Table 1 briefly explores the current literature available across various academic fields (i.e., business, psychology, physiology, injury & health, computer sciences, and coaching) to evidence the terminology used.

## 4. Critique of current terminology

When examining Table 1, it is clear that many papers interchangeably use the terms athletes, competitors, and players when discussing esports. This further highlights the need for clarity within the terminology, with considerations taken to define what is considered aligned to an esports athlete and who is encapsulated when discussing the casual gamer. Secondly, out of the sixteen papers outlined within the table, only four papers explicitly define their population, further emphasizing the need to understand the specific distinctions between them.

## 5. Discussion

We argue that esports athlete (or e'athlete as an abbreviation) is a suitable term that encapsulates individuals who compete in any esports to achieve an in-game ranking or who compete in a formalized competition. Esports athlete (e'athlete) should be used similarly to the umbrella term “athlete”, broadly referring to the players of all types of sports (25). Furthermore, the term “player” can be used when referring to players within a specific esports title [e.g., LoL player, Counter-Strike: Global Offensive (CS:GO) player]. Standardising this terminology across each academic discipline will help differentiate and offer clarification between esports-specific research (competitive games) and video game research (non-competitive games).

An example comes from studies by Trotter et al. (19, 26). In their 2020 study, they compare the health and psychical activity behaviours of e'athletes with those of normative data. Future research could compare e'athletes with gamers, athletes, or normative values. Similarly, the study by Trotter et al. (11) explored the efficacy of a school-based esports program. Future studies could examine how e'athletes would benefit from esports-specific interventions to enhance health behaviours (e.g., physical activity; see 27).

The aim of this piece was to briefly discuss the various terms used in academic research to describe esports participants and highlight the need for consistent terminology across academic research. The absence of a cohesive naming convention or terminology poses a challenge within the field, hindering researchers' ability to consolidate existing literature and derive significant, cross-study conclusions. This issue becomes particularly evident when conducting systematic reviews or meta-analysis, where the lack of a unified approach complicates the synthesis process.

Looking towards sporting research, the use of “athlete” is generally a broader term used to describe athletes across any range of sports, however, in sports, athletes are often categorized by their level of eliteness (28), expertise (29) and competition level (i.e., national/international, county, university, and club; see (25). However, due to the infancy of the esports industry and esports research, no guidelines exist to help delineate between eliteness, expertise, or competition standards, which leads to the generalization of populations within the ranking system. Further research is still required to understand the appropriate adjectives used to delineate the expertise of esports athletes (i.e., amateur, semi-professional, professional and elite), which can help researchers be more specific within their sampling across the various disciplines that have started to investigate esports.
